# Intra-articular corticosteroid utilization and characterizations of use in juvenile idiopathic arthritis within the PR-COIN registry

**DOI:** 10.3389/fped.2024.1423362

**Published:** 2024-07-23

**Authors:** Erin Balay-Dustrude, Jennifer E. Weiss, Y. Ingrid Goh, Nathan Rubin, Danielle R. Bullock

**Affiliations:** ^1^ Pediatric Rheumatology, Department of Pediatrics, Seattle Children’s Hospital, University of Washington, Seattle, WA, United States; ^2^ Seattle Children’s Hospital and Research Center, Seattle, WA, United States; ^3^ Pediatric Rheumatology, Department of Pediatrics, Hackensack University Medical Center, Hackensack Meridian School of Medicine, Hackensack, NJ, United States; ^4^ Division of Rheumatology, The Hospital for Sick Children, Toronto, ON, Canada; ^5^ Child Health Evaluative Sciences, SickKids Research Institute, Toronto, ON, Canada; ^6^ Biostatistics Core, Masonic Cancer Center, University of Minnesota, Minneapolis, MN, United States; ^7^ Pediatric Rheumatology, Allergy, and Immunology, Department of Pediatrics, M Health Fairview Masonic Children’s Hospital, University of Minnesota, Minneapolis, MN, United States

**Keywords:** juvenile idiopathic arthritis, corticosteroid injection, treatment, outcome, registry

## Abstract

**Objective:**

Intra-articular corticosteroid injections (IACI) have been shown to be effective at improving arthritis across juvenile idiopathic arthritis (JIA) categories. The American College of Rheumatology (ACR) recommends IACI use as primary and adjunctive therapy for JIA patients. However, there remains minimal data describing actual IACI use in North America. The objective of this study was to describe and to evaluate IACI use in JIA, utilizing the Pediatric Rheumatology Care and Outcomes Improvement Network (PR-COIN) registry.

**Methods:**

Study participants from 13 sites were enrolled in the PR-COIN registry from 2011 to 2015. Demographic and clinical variables were summarized and Chi-squared and *t*-tests were used to evaluate differences between participants who did or did not receive IACI. Multiple logistic regression models were used to evaluate characteristics associated with IACI treatment.

**Results:**

Our study included 3,241 participants, the majority of whom were white (85%), female (71%) and had oligoarticular JIA (39%). IACI was administered at least once in 23% of participants, the majority of whom had oligoarticular disease (52.5%), but overall use in oligoarticular participants was low at 30.8%. IACI use varied significantly between treatment centers and use was associated with oligoarticular disease, ANA positivity, and use of other systemic medications.

**Conclusion:**

This study demonstrates that participants with JIA enrolled in the PR-COIN registry between 2011 and 2015 with persistent oligoarticular disease, ANA positivity, and use of other systemic medications were more likely to receive IACI. However, IACI use was lower than expected for oligoarticular participants.

## 1 Introduction

Juvenile idiopathic arthritis (JIA) is one of the most common chronic rheumatic diseases of childhood, with a prevalence of approximately 1 per 1,000 population (1). The International League of Associations for Rheumatology (ILAR) classification includes seven JIA subtypes. The most common subtype, oligoarticular disease (≤4 joints involved), accounts for 50%–80% of all chronic arthritis cases in North America and Europe (1). Expeditious and effective treatment of JIA is required in order to relieve pain, promote growth, and prevent permanent functional disabilities and joint destruction (2, 3).

A variety of treatment modalities are available for use by the pediatric rheumatologist to arrest the inflammatory process and achieve disease control. The 2011, 2013, 2019 and 2021 American College of Rheumatology (ACR) JIA treatment guidelines have consistently recommended intra-articular corticosteroid injections (IACI) as a primary treatment for oligoarticular disease and as adjunct or bridging therapy for polyarticular disease, sacroiliitis, and systemic disease. IACI are often used in combination with other therapeutics including non-steroidal anti-inflammatory drugs (NSAIDs), conventional synthetic disease modifying antirheumatic drugs (csDMARDs), and biologic DMARDs (2–5). The variety of treatment options and lack of evidence to specifically recommend one treatment over another, which is highlighted by the ACR guidelines, has resulted in varying strengths of practice recommendations, and likely varying levels of adherence to these recommendations.

Evidence of the effectiveness of IACI in the treatment of JIA is based on a number of retrospective and prospective studies which define effectiveness as a prolonged period of inflammatory inactivity in the injected joint after treatment (6–10). However, achievement of disease inactivity and the duration of efficacy after IACI varies depending on JIA subtype, age, disease duration, antinuclear antibody (ANA) positivity status, concomitant systemic therapy, and preparation of intra-articular glucocorticoid used (7, 10–14). Furthermore, ACR guidelines and multiple studies demonstrate favor triamcinolone hexacetonide (TH) over triamcinolone acetonide (TA), as it has been shown to induce longer periods of remission in injected joints (4, 5, 9, 15–17). In two fairly recent studies, the response rate to IACI, which was defined as absence of arthritis at 6 months, ranged from∼50%–70% (9, 10). It would be expected, then, that many children require other therapies beyond IACI.

While these studies provide a foundation for treatment, and the initial 2011 ACR guidelines recommended IACI use, there is limited data on the actual clinical context in which IACI are utilized and the prevalence of use in a large population of JIA patients. Given this knowledge gap, the goal of this study was to evaluate the baseline use of IACI in the treatment of JIA in a large North American (United States and Canada) cohort. We examined the prevalence and predictors of IACI use in participants with JIA who were enrolled in the Pediatric Rheumatology Care and Outcomes Improvement Network (PR-COIN) registry between January 1, 2011, and July 31, 2015.

PR-COIN is a multicenter “Learning Network” in North America that uses quality improvement methods to develop and evaluate JIA management strategies, with a goal of improving disease outcomes for children with JIA. The network focuses on collection of data that can be used at the point of care to inform treatment decisions, with an emphasis on close partnership with patients and their families. Implementation of interventions proven to improve chronic illness care – for example pre-visit planning, population management, and shared decision making – and how such interventions impact disease outcomes is a priority (18). While PR-COIN has an established goal to administer IACI in a timely fashion, within 2 weeks of identified need, there has not yet been a network-wide initiative around the use of this treatment modality. This study, then, serves to shed light on baseline treatment patterns within the network. It helps identify known but important gaps in data collection and helps frame the need for future work on how treatment decisions ultimately impact outcomes.

## 2 Methods

### 2.1 Population

De-identified data for all children with JIA enrolled in the PR-COIN registry from 2011 inception through July 2015 were extracted and analyzed. Any patient with JIA at a participating PR-COIN center was eligible for enrollment in the registry though the actual process of enrollment varies by center. For example, some centers require patient consent to enroll while others do not. Notably, registry enrollment occurred between 2011 and July 2015 though participants may have been diagnosed or received treatments prior to their enrollment. Treatment information reported reflects treatments administered during this time frame from 2011 to 2015, or prior to enrollment when reported. Because data was de-identified, analysis and results could not include specific dates as only days/months from an unknown referent date were provided.

This project was approved by the Institutional Review Board (IRB) and not considered human subjects research. Data included patient demographics, ILAR subtype, diagnosis and encounter dates (listed as days from referent point), ANA positivity status, and treatments. Treatments captured included: any NSAID, IACI (triamcinolone acetonide, triamcinolone hexacetonide, and other), non-biologic DMARDs (azathioprine, cyclosporine, hydroxychloroquine, leflunomide, methotrexate, sulfasalazine, and other) and biologics (abatacept, adalimumab, anakinra, canakinumab, certolizumab, etanercept, golimumab, infliximab, rilonacept, rituximab, tocilizumab, and other). Data from participants enrolled at 12 out of 13 eligible medical centers were analyzed; one center did not have IACI data available and was therefore excluded. For some participants who had discrepant diagnosis dates, the earliest reported date was used.

### 2.2 Statistical analysis

Demographic and clinical factors were analyzed for differences between those who received at least one IACI and those who did not receive any IACI using Chi-square tests and *t*-tests for categorical and continuous measures, respectively. Multiple logistic regression models, along with odds ratios and 95% confidence intervals, were used to investigate demographic or disease features associated with the odds of receiving IACI. Site, age at diagnosis, ANA status, ILAR code, race, ethnicity, NSAID, DMARD, and biologic DMARD use were selected *a priori* as relevant factors to be included in the model. Some participants received more than one IACI, so analysis was run using both minimum and maximum assumptions for administration, meaning that when there was a report of IACI use in the data, but no date was listed, the minimum assumption was that the missing date was one of the subject's other non-missing date values. The maximum assumption was that each missing date was assumed to be a unique date. As these results were without significant differences, the minimum IACI use assumption results were reported. When there were missing, unknown, or incomplete data, these were excluded from the regression model. R (R Core Team) Version 4.0 was used for all analyses. *P*-values less than 0.05 were considered statistically significant.

## 3 Results

### 3.1 Registry data and population demographics

There were 3,241 participants enrolled in the PRCOIN registry from 2011 to 2015 and included in the analysis, with 14 participants excluded for incorrect or missing data. The majority of registry participants were from the United States (81.5% vs. 18.5% from Canada), White (85%), female (71%), and had oligoarticular disease (39%, persistent and extended), similar to previously reported North American characteristics of participants with JIA (Table 1) 0/0/00 0:00:00 AM (19, 20). Persistent oligoarticular participants were defined as those with ≤4 joints involved, while extended oligoarticular participants were those whose joint count extended to involvement of >4 joints after the first 6 months of their disease. There was some variability of ILAR subtype by site (Supplementary Table S1). Patient duration in the registry varied, with 23.6% (765/3,241) having only one visit recorded, 29.5% (956/3,241) enrolled in the registry for less than a year, and 46.9% (1,519/3,241) enrolled for a year or more. Time from diagnosis to first registry encounter was 48.4, 48 months (mean, SD), meaning many patients were enrolled well into their diagnosis.

**Table 1:** General demographic and disease/treatment features of PR-COIN registry participants from 2011 to 2015.

Characteristic	*N* = 3,241*
Age at diagnosis	
Mean age in years (SD)	7.6 (4.8)
Months between diagnosis and 1st registry visit	
Mean duration in months (SD)	48.4 (48.0)
Duration in registry	765 (23.6%)
1 visit	956 (29.5%)
<1 year	1,519 (46.9%)
≥1 year	
Gender	
Female	1,937 (70.9%)
Male	795 (29.1%)
Race	
Non-White	388 (15.3%)
White	2,149 (84.7%)
Ethnicity	
Hispanic	291 (11.7%)
Non-Hispanic	2,192 (88.3%)
ILAR Code	
Oligoarticular persistent	993 (30.7%)
Oligoarticular extended	276 (8.5%)
Polyarticular RF (+)	172 (5.3%)
Polyarticular RF (-)	883 (27.3%)
Psoriatic arthritis	219 (6.8%)
Enthesitis related	398 (12.3%)
Systemic	208 (6.4%)
Undifferentiated	84 (2.6%)
Country	
US	599 (18.5%)
Canada	2,641 (81.5%)
IACI use^	
TH	513 (15.8%)
TA	300 (9.3%)
Other	23 (0.7%)
None	2,494 (77%)
Any IACI use	747 (23%)
Any NSAID use	2,049 (63.2%)
Any DMARD use	1,739 (53.7%)
Any biologic use	1,495 (46.1%)

SD, standard deviation; RF, rheumatoid factor; US, United States; IACI, intra-articular corticosteroid injection; TH, triamcinolone hexacetonide; TA, triamcinolone acetonide; ILAR, international league against rheumatism; IACI, intra-articular corticosteroid injection; NSAID, non-steroidal anti-inflammatory drug; DMARD, disease modifying anti-rheumatic drug.

* Some values may not add up to total due to missing data.

^ Some patients received more than one type of IACI.

### 3.2 IACI Use

Twenty-three percent (747/3,241) of participants received one or more IACI, with TH being the corticosteroid formulation used most often, accounting for 61.3% of IACIs given (513/836). The median time to first captured injection for those who received IACI treatment was 28 months (range 25–230 months) after diagnosis. Retrospective treatment data was not required for registry enrollment so there are likely instances of IACI prior to what was captured. Negative values reflect IACIs received before participants were enrolled in the registry but after their initial diagnosis. IACI was the least commonly used treatment, with other treatments (NSAIDs, DMARDs, and biologics) being used in a greater percentage of participants overall (Table 1), keeping in mind that individual participants may receive multiple therapies either in sequence or concurrently. Data for sequence of medication, or multiple medications administered within the same class, for example repeat IACI use, was limited and incomplete so was not included in this analysis.

The majority of IACI were administered in participants who had oligoarticular disease, which accounted for 52.5% (391/745) of the IACI use (Table 2). However, considering that IACI is a main treatment modality recommended in this subtype, then the overall use in participants with oligoarticular disease remained low, with only 30.8% (391/1,269) of participants with oligoarticular disease receiving IACI. This indicates that 69.2% (878/1,269) either did not receive IACI therapy or that this was not captured. For participants with polyarticular disease (rheumatoid factor positive and negative), 21.6% (228/1,055) received IACI, followed by 18.7% (41/219), 13% (42/398), 10.5% (22/208), and 13% (11/84) for patients with psoriatic arthritis, enthesitis related arthritis, systemic JIA, and undifferentiated disease respectively.

**Table 2:** Comparison of features for patients who did vs. did not receive intra-articular corticosteroid injections.

Characteristic	IACI use - yes (*N* = 747^)	IACI use - no (*N* = 2,494^)	*P*-value
Center**			<0.001*
Country			0.525
United States	614 (82.3%)	2,027 (81.3%)	
Canada	132 (17.7%)	467 (18.7%)	
Region			<0.001*
Canada (2 sites)	132 (17.%)	467 (18.7%)	
US Midwest (2 sites)	206 (27.6%)	452 (18.1%)	
US Northeast (4 sites)	301 (40.3%	1,035 (41.5%)	
US South (4 sites)	77 (10.3%)	270 (10.8%)	
US West (4 sites)	30 (4.0%)	270 (10.8%)	
Duration in registry			<0.001*
One visit	64 (8.6%)	701 (28.1%)	
<1 year	186 (24.9%)	770 (30.9%)	
≥1 year	497 (66.5%)	1,022 (46.9%)	
Age at diagnosis (years)			0.008*
Mean (SD)	7.1 (4.7)	7.7 (4.9)	
Range	0.7–17.9	0.2–23.7	
Gender			0.012*
Female	469 (74.9%)	1,468 (69.7%)	
Male	157 (25.1%)	638 (30.3%)	
Race			0.004*
Non-White	69 (11.6%)	319 (16.4%)	
White	525 (88.4%)	1,624 (83.6%)	
Ethnicity			0.165
Hispanic	58 (10.1%)	233 (12.2%)	
Non-Hispanic	517 (89.9%)	1,675 (87.8%)	
ILAR Code			<0.001*
Oligoarticular persistent	311 (41.7%)	682 (27.4%)	
Oligoarticular extended	80 (10.7%)	196 (7.9%)	
Polyarticular RF (+)	33 (4.4%)	139 (5.6%)	
Polyarticular RF (-)	195 (26.2%)	688 (27.7%)	
Psoriatic	41 (5.5%)	178 (7.2%)	
Enthesitis related	52 (7.0%)	346 (13.9%)	
Systemic	22 (3.0%)	186 (7.5%)	
Undifferentiated	11 (1.5%)	73 (2.9%)	
ANA status			<0.001*
Positive	439 (58.8%)	1,104 (44.3%)	
Negative	281 (37.6%)	1,207 (48.4%)	
Unknown/missing	27 (3.6%)	183 (7.3%)	
Any NSAID use	594 (79.5%)	1,455 (58.3%)	<0.001*
Any DMARD use	465 (62.2%)	1,274 (51.1%)	<0.001*
Any biologic use	355 (47.5%)	1,140 (45.7%)	0.383

SD, standard deviation; RF, rheumatoid factor; US, United States. IACI, intra-articular corticosteroid injection; ILAR, international league against rheumatism; ANA, antinuclear antibody; NSAID, non-steroidal anti-inflammatory drug; DMARD, disease modifying anti-rheumatic drug.

^ Individual categories may not add up to this number due to missing data.

* Statistically significant difference for characteristic.

** Center specific IACI use available in Supplementary Table S2.

There were statistically significant differences among those who did vs. did not receive IACI by center, indicating that treatment practices may vary among clinical sites (Table 2). For example, less than 10% (5/51) of those at site B received an IACI compared to just over 30% (145/475) at site A (Supplementary Table S2). There was no statistically significant difference between sites based on country (United States vs. Canada). When centers were grouped by region, there was a detectable difference, with centers in the United States Midwest demonstrating the highest rate of IACI use (206/658 = 31.3%) and US western centers demonstrating the lowest use (30/300 = 10.0%) (Table 2).

There were also notable differences for IACI use based on duration in the registry. Participants with only one visit recorded in the registry (64/765 = 8.4%) or less than 1 year of registry participation (186/956 = 19.5%) demonstrated lower IACI use compared to participants in the registry for a year or more (497/1,519 = 32.7%) (Table 2).

### 3.3 Characteristics of IACI recipients

There were statistically significant differences for IACI use by patient ILAR subtype, gender, race, and ANA status (Table 2). The use of NSAIDs and DMARDs was greater among those who received IACI as compared to those who did not, while biologic use between the groups was similar.

Similarly, when accounting for these various characteristics through a logistic regression model, we found that treatment center, ILAR category, ANA positivity, and use of other systemic medications were associated with greater odds of receiving IACI (Table 3). Participants with persistent oligoarticular disease expectedly had the highest odds of receiving IACI treatment, when adjusting for all the other factors in the model.

**Table 3:** Logistic regression *p*-values and odds ratios for significant comparisons.

Parameter	Odds ratio	95% confidence interval	*p*-value
Center^			<0.001*
Age at diagnosis			0.20
Gender			0.57
Race			0.29
Ethnicity			0.54
ANA status			0.011*
Positive vs. negative	1.31	1.06–1.63	
ILAR code			<0.001*
Oligoarticular persistent	ref	–	
Oligoarticular extended	0.64	0.43–0.93	
RF + polyarticular	0.36	0.20–0.57	
RF - polyarticular	0.41	0.31–0.54	
Psoriatic	0.36	0.21–0.53	
Enthesitis-related	0.24	0.15–0.35	
Systemic	0.20	0.14–0.44	
Undifferentiated	0.24	0.09–0.52	
NSAID use			<0.001*
Yes vs. no	2.61	2.03–3.39	
DMARD use			0.002*
Yes vs. no	1.35	1.08–1.93	
Biologic use			<0.001*
Yes vs. no	1.52	1.20–1.93	

Reference group use for ILAR code associations: Persistent Oligoarticular.

RF, rheumatoid factor; ANA, antinuclear antibody; ILAR, international league against rheumatism; NSAID, non-steroidal anti-inflammatory drug; DMARD, disease modifying anti-rheumatic drug.

^ Specific comparisons and odds ratios not included here due to multiple possible pairwise comparisons.

* Statistically significant comparison.

## 4 Discussion

The goals of treatment for patients with JIA encompass the use of safe, timely, and effective medication to achieve disease control. Ultimately, understanding which therapies are most effective for which patients will inform medical decision-making and lead to improved clinical outcomes. Achieving this goal requires rigorous comparative effectiveness studies, designed in an informed, data driven manner. This study, a secondary analysis of existing data from the PR-COIN registry, is a first step in understanding how IACI are being used in clinical practice and whether there are predictors of their use. This, in turn, can help shed light on what other factors that may contribute to use should be captured and evaluated in future studies.

In this study, we observed that prevalence of IACI use varied by treatment center. When comparing centers in the United States vs. Canada, we did not observe a difference in use; however, when divided further to include regions within the United States, there were observed differences. This may indicate that therapy decisions are driven less by insurance coverage or availability of medication, for example, as these would likely differ more between countries. Rather, perhaps there are factors driven by local culture, number of providers at a center, availability of ancillary services such as pediatric anesthesia and child life specialists, and whether the hospital is a teaching/training institution.

Our cohort, which includes prevalent cases of JIA enrolled in the PR-COIN registry between 2011 and 2015, captures a time period of IACI use prior to and during the early stages of the ACR guidelines for IACI use. Given the mean time from disease diagnosis to registry enrollment (48.4 months), treatments administered shortly after diagnosis, which may well have been IACI use, were often not captured in the registry with enough detail to evaluate in this analysis. Thus, it is not unexpected that we observed IACI use to be overall lower than recommended by the ACR guidelines, particularly in those with oligoarticular disease. We also found that other medications including NSAIDs, biologics and conventional DMARDs were associated with treatment with IACI, which we interpret to mean that within this cohort, IACI were not the primary treatment modality in most cases but rather an adjunct therapy. This may indicate that IACI do not necessarily alleviate the need for additional rheumatologic treatments to achieve disease control but are used often as bridging or adjunct therapy, a concept that is supported by other studies (9, 10). This consideration is further corroborated by Papadopouluo et al., who demonstrated that IACI may be utilized as bridge therapy while awaiting systemic therapy to take effect and have been shown to be effective even in polyarticular patients with the benefit of avoiding or limiting systemic corticosteroid therapy and its side effects (21).

Regarding selection of injectable corticosteroid medication, in the PR-COIN registry, TH was used at a higher rate than TA. This finding likely indicates a preference for this medication when available, as our data represents a time period when TH was still widely available in the US, prior to its discontinuation in 2015. These findings are in line with the available literature, both historical and recent, which shows a clear benefit in terms of longevity of efficacy for IACI with TH over TA (9, 17, 22). Given the clinical superiority of TH, one might question why it did not account for an even greater percent of IACI in our cohort.

Further, we sought to understand which participants received IACI most frequently in the PR-COIN registry. In this study, we observed differences in recipients of IACI by ILAR code, gender, race and ANA status. This matches what might be expected, with use being higher for oligoarticular disease, more common among females and those who are white, which accounts for most patients with JIA and in particular those with oligoarticular disease, and among those who are ANA positive, with ANA positivity being most common in the oligoarticular subtype. Using a logistic regression model, we identified potential predictors of IACI use which included: treatment center, ILAR category, and ANA positivity. This similarly aligns with what we would expect.

Finally, the majority of the available literature surrounding IACI use focuses on predictors of disease course after injection. However, there has been limited evaluation of predictive factors for the use of IACI as a first line of treatment in JIA, despite evidence to suggest that IACI were most effective for young JIA patients with a short disease course (20). Thus, further evaluation of which patients are most likely to both receive and benefit from IACI early in the disease course is warranted. This gap in the literature, in combination with our study results, suggest there may be under-utilization of IACI in this population of JIA patients, and advocates for the increased use of IACI early in the disease course, whether as mono-therapy or in combination with other treatment modalities.

The PR-COIN registry during the study period encompassed 13 centers with a focus on care and outcomes improvement. Considering the generalizability of our findings, the demographic characteristics of our cohort are similar to demographics of other large North American databases, demonstrating that patient enrollment is likely representative of the JIA population (23). However, given the time period of the study, this dataset may under-represent medication use including IACI, as data collection of this type was not a primary focus of the registry at that time. The network emphasizes using data collected at the point of care, with data collection being performed on a voluntary basis. As such, data elements that directly capture outcomes such as the patient and provider assessment of disease activity are prioritized. While medication data such as IACI use is also captured, there are known gaps in the completeness of this data within the registry. Given that data entry and enrollment practices differ among centers, such variability could account for some of our findings.

It is also important to acknowledge that actual treatment decisions may differ from guidelines due to a number of factors, including patient/family preferences and systems of care. For many pediatric patients, joint injections are performed under sedation so availability of sedation and space and time allocation for rheumatology procedures could be a factor. There may be center or regional differences in who is trained to perform injections. Ultimately, then, how closely actual treatments mirror what might be expected can be influenced by a number of factors beyond treatment guidelines. Such factors would ideally be captured in future work on this subject.

Since 2015, the PR-COIN network has continued to grow and has expanded efforts for data collection, incorporating standardized practices with electronic medical record (EMR) integration, which in the long term is likely to lend to a more clear understanding of medication use and potential associations to patient disease outcomes. This creates an opportunity for future research endeavors utilizing this robust registry. It also allows opportunity for collaboration. PR-COIN does not specifically intend to perform robust comparative effectiveness trials related to medications, though such trials are certainly needed to understand what treatment modalities might result in the best outcomes. PR-COIN is situated to help shed light on the gaps that need to be better studied and also to ultimately understand how implementation of recommended treatment strategies and quality improvement initiatives might affect outcomes over time.

### 4.1 Limitations

A notable shortcoming of this study is selection bias. Only patients with JIA who were enrolled in the PR-COIN registry were included, and it is possible that these patients are not representative of all patients with JIA. Additionally, there is very likely variability in registry enrollment patterns and data completeness between sites. At some centers, all providers help enroll patients while at other centers it may be only a limited number of providers who enroll their patients. Enrollment and data entry at the time of this study was primarily done through manual extraction, and there is no available information to capture what percent of the total JIA population from participating centers is represented by enrollment during this time frame. Furthermore, as previously noted, during this study period instances of IACI use for those in the registry may have been missed. This could be because IACI were given prior to registry enrollment, without retrospective treatment data necessarily being entered, and/or because only a short part of the patient course was captured in the registry, with over half of participants having been enrolled for less than a year. In any disease cohort, capturing the entirety of their course, including treatment given prior to enrollment and disease outcomes after treatment is an ideal but often difficult to achieve goal. Again, comprehensive, longitudinal treatment data and specific outcomes related to each treatment modality was not the primary objective for PR-COIN, though is of course desirable. This data set has additional limitations including lack of documentation of which joints were affected and/or injected and whether the same joint was injected repeatedly, which would lend to the depth and understanding of IACI use across the patient disease course. Further, limitations in available start dates for the various treatment modalities limited our ability to understand the relationships between treatments and their impact on disease control. Presumably, if a patient had well-controlled disease then joint injections and/or other treatment modifications would not be needed and so perceived underutilization of treatments could stem from our inability to determine disease activity and disease outcomes in relationship to treatment in this population.

In July 2015, PR-COIN transitioned to a new registry platform, creating some discontinuity in data. We therefore chose to analyze data only from the initial registry. Furthermore, in 2015 the production and availability of TH in the United States was discontinued; thus, comparing use of IACI before and after this time period could create limits in interpretation of IACI use and efficacy. We would predict a decline in IACI use in general after 2015. Another shortcoming, inherent to secondary analysis of existing data, is the fact that there may be additional confounding covariates that are not accounted for in the current data set.

### 4.2 Future directions

The PR-COIN network has continued to grow and expand since the period evaluated in this analysis and now encompasses over 8,500 patients with JIA. Transition to a new registry platform occurred in late 2022, with aspirations for more complete EMR integration. There are improvement efforts underway which will allow for more robust, detailed, and equitable data collection. As the network continues to focus on patient outcomes, standardization, and guideline-based care practices, this will allow for deeper examination of the relationship between treatment selection and duration of treatment in relation to clinical outcomes. In light of PR-COIN's focus on patient engagement, it will be important to consider how demonstration of treatment efficacy may influence treatment decisions. For example, patients and parents might wish to avoid systemic therapies when working to obtain disease control. Thus, a better and more direct understanding of the effectiveness of IACI compared to other treatments could influence a patient's acceptance of and adherence to recommended treatments. Further, a comparative analysis of historical and current IACI use practices may allow for deeper understanding of treatment practice changes over time and any potential relationship to patient disease outcomes.

## 5 Conclusion

In summary, this study highlights that the PR-COIN registry is a suitable representation of the North American JIA population, and analysis of registry data is able to provide meaningful insights on disease treatments utilized in patients with JIA. Within the registry from 2011 to 2015 we found that patients with persistent oligoarticular disease, ANA positivity, and use of other systemic medications were more likely to receive IACI. Utilization patterns also varied by treatment center. Overall, prevalence of IACI use was lower than might be expected, in particular for those with oligoarticular disease, though the timing of our study in relation to published guidelines needs to be considered. Our findings highlight variability in treatment and that there are likely multiple factors contributing to treatment decisions throughout the disease course. Ultimately, understanding how these treatment decisions impact outcomes and whether standardization results in better outcomes is imperative. PR-COIN is well situated to shed light on this moving forward.

## Data Availability

The raw data supporting the conclusions of this article will be made available by the authors, without undue reservation.
