# Supplementary material for: Intra-articular corticosteroid utilization and characterizations of use in juvenile idiopathic arthritis within the PR-COIN registry
Source: Front Pediatr. 2024 Jul 23;12:1423362. doi: 10.3389/fped.2024.1423362 (PMC11300215; doi:10.3389/fped.2024.1423362)
Supplement: Supplementary file 1 [file Table1.docx]

Supplementary Material

| Supplementary Table 1: Participant ILAR code by PR-COIN registry site | | | | | | | | | | | |  |  |  |
| --- | --- | --- | --- | --- | --- | --- | --- | --- | --- | --- | --- | --- | --- | --- |
|  | **A**  **(N=475)** | **B**  **(N=51)** | **C**  **(N=207)** | **D**  **(N=360)** | **E**  **(N=194)** | **F**  **(N=249)** | **G**  **(N=91)** | **H**  **(N=322)** | **I**  **(N=140)** | **J**  **(N=461)** | **K**  **(N=183)** | | **L**  **(N=508)** | **Total**  **(N=3241)** |
| **ILAR Code** |  |  |  |  |  |  |  |  |  |  |  | |  |  |
| Systemic JIA | 25 (5.3%) | 4  (7.8%) | 11 (5.3%) | 26 (7.2%) | 8  (4.1%) | 14 (5.6%) | 3 (3.3%) | 16 (5.0%) | 12 (8.6%) | 27 (5.9%) | 16 (8.7%) | | 46 (9.1%) | 208  (6.4%) |
| Polyarticular  RF (-) | 152 (32.2%) | 20 (39.2%) | 64 (30.9%) | 101 (28.1%) | 37 (19.1%) | 60 (24.1%) | 34 (37.8%) | 82 (25.5%) | 36 (25.7%) | 97 (21.0%) | 52 (28.4%) | | 148 (29.4%) | 883 (27.3%) |
| Polyarticular  RF (+) | 24 (5.1%) | 7 (13.7%) | 16 (7.7%) | 26 (7.2%) | 6  (3.1%) | 17 (6.8%) | 4 (4.4%) | 21 (6.5%) | 4  (2.9%) | 17 (3.7%) | 14 (7.7%) | | 16 (3.2%) | 172  (5.3%) |
| Oligoarticular persistent | 120 (25.4%) | 12 (23.5%) | 73 (35.3%) | 123 (34.2%) | 55 (28.4%) | 76 (30.5%) | 27 (30.0%) | 135 (41.9%) | 68 (48.6%) | 110 (23.9%) | 40 (21.9%) | | 154 (30.6%) | 993 (30.7%) |
| Oligoarticular extended | 31 (6.6%) | 4  (7.8%) | 14 (6.8%) | 28 (7.8%) | 16 (8.2%) | 14 (5.6%) | 7 (7.8%) | 26 (8.1%) | 6  (4.3%) | 43 (9.3%) | 23 (12.6%) | | 64 (12.7%) | 276  (8.5%) |
| Psoriatic | 58 (12.3%) | 3  (5.9%) | 10 (4.8%) | 18 (5.0%) | 11 (5.7%) | 15 (6.0%) | 6 (6.7%) | 14 (4.3%) | 9  (6.4%) | 42 (9.1%) | 8  (4.4%) | | 25 (5.0%) | 219  (6.8%) |
| Enthesitis Related | 61 (12.9%) | 1  (2.0%) | 18 (8.7%) | 30 (8.3%) | 41 (21.1%) | 50 (20.1%) | 9 (10.0%) | 26 (8.1%) | 5  (3.6%) | 110 (23.9%) | 7  (3.8%) | | 40 (7.9%) | 398 (12.3%) |
| Undifferentiated | 1  (0.2%) | 0  (0.0%) | 1  (0.5%) | 8  (2.2%) | 20 (10.3%) | 3  (1.2%) | 0 (0.0%) | 2  (0.6%) | 0  (0.0%) | 15 (3.3%) | 23 (12.6%) | | 11 (2.2%) | 84  (2.6%) |
| Missing (n) | 3 | 0 | 0 | 0 | 0 | 0 | 1 | 0 | 0 | 0 | 0 | | 4 | 8 |
| Registry participant ILAR category by PR-COIN registry site, n (%). Site identifier codes coded for privacy, available upon request.  RF: rheumatoid factor, ILAR: International League Against Rheumatism | | | | | | | | | | | | | | |

| Supplementary Table 2: IACI use by center | | |
| --- | --- | --- |
| **Center** | **IACI Use - Yes**  **(N = 747)** | **IACI Use - No**  **(N = 2494)** |
| A | 145 (19.4%) | 330 (13.2%) |
| L | 121 (16.2%) | 387 (15.5%) |
| J | 118 (15.8%) | 343 (13.8%) |
| D | 89 (11.9%) | 271 (10.9%) |
| H | 74 (9.9%) | 248 (9.9%) |
| K | 61 (8.2%) | 122 (4.9%) |
| C | 40 (5.4%) | 167 (6.7%) |
| I | 37 (5.0%) | 103 (4.1%) |
| F | 25 (3.3%) | 224 (9.0%) |
| E | 21 (2.8%) | 173 (6.9%) |
| G | 11 (1.5%) | 80 (3.2%) |
| B | 5 (0.7%) | 46 (1.8%) |
| Participant receipt of IACI by specific PR-COIN Registry site, n (%) | | |
